# Effect of Nd:YAG Laser Surface Pretreatments and Bonding Protocols on Shear Bond Strength of Monolithic Zirconia with Varying Yttria Contents to Composite Resin

**DOI:** 10.1055/s-0045-1808260

**Published:** 2025-05-07

**Authors:** Ahmed Yaseen Alqutaibi, Ahmad Abdulkareem Alnazzawi, Mohammed H. AbdElaziz, Ahmed E. Farghal, Mohamed F. Aldamaty, Mohammed Ahmed Alghauli

**Affiliations:** 1 Substitutive Dental Science Department, College of Dentistry, Taibah University, Medina, Saudi Arabia; 2 Prosthodontics Department, Faculty of Dentistry, Ibb University, Ibb, Yemen; 3 Fixed Prosthodontics Department, Faculty of Dental Medicine, Al-Azhar University, Cairo, Egypt; 4 Department of Restorative and Aesthetic Dentistry, College of Dentistry, Almaaqal University, Basrah, Iraq

**Keywords:** 3Y-TZP, 5YSZ, bond strength, dental ceramic, Nd:YAG laser, surface treatment

## Abstract

**Objectives:**

This study aimed to evaluate the impact of different surface pretreatments and bonding protocols on the shear bond strength (SBS) of two monolithic zirconia materials to composite resin.

**Material Methods:**

A total of 200 zirconia specimens, 3Y-TZP ( *n* = 100) and 5YSZ ( *n* = 100), were allocated into five groups: Control with no treatment, air-particle abrasion (APA), Nd:YAG (neodymium-doped yttrium aluminum garnet) laser treatment (L), a combination of APA and L, and laser treatment followed by cold plasma (CAP). Half of the specimens received a primer application before bonding with resin cement. Surface morphology was assessed using scanning electron microscopy, and SBS testing was conducted with a universal testing machine.

**Statistical Analysis:**

The SBS analysis was done using multiway analysis of variance ( *p* ≤ 0.05).

**Results:**

Different surface pretreatments and 10-methacryloyloxydecyl dihydrogen phosphate primer application significantly increased SBS values ( *p* ≤ 0.001). APA was associated with the highest SBS values, followed by APA + laser and laser + CAP. However, the combination of APA with L slightly reduce the bond strength. While the application of laser alone possesses the lowest SBS among the surface pretreatment methods, the control group was the worst by far. Different zirconia materials showed no impact on SBS values.

**Conclusion:**

APA surface pretreatment might still be the gold standard for zirconia adhesion. Laser surface pretreatment is a viable, less destructive option. Combining APA with laser slightly reduces SBS, while combining two inert surface pretreatment methods, such as laser and CAP, leads to enhancement of SBS compared with laser alone. Zirconia primer is highly recommended for bonding protocol. No special considerations should be taken for different yttria contents, as both materials reported comparable bond strength within the same coupled variables.

## Introduction

All-ceramic dental restorations are becoming increasingly popular among patients and widely recommended by dentists. Their long-term success depends on strong adhesion to the underlying structures. 1 Achieving a reliable bond requires selecting the right luting protocol and preparing the surface to maximize adhesive potential. Proper surface pretreatments and bonding protocols improve bonding strength, ensuring long-lasting results. 1 2 3

Different ceramic materials require specific surface pretreatments. Glass ceramics are best treated with 5% hydrofluoric acid etching, which effectively modifies the glass structure. 4 In contrast, oxide ceramics like monolithic zirconia are chemically inert and need mechanical roughening. The most common method is air-borne particle abrasion (APA), which has proven successful in both clinical 2 5 and laboratory studies. 5 6 However, APA can introduce stress into the zirconia structure, potentially leading to microcracks and phase transformations. 7

To address these concerns, researchers have explored alternative surface pretreatments with less impact on zirconia. 7 8 9 10 11 12 One promising approach is laser surface irradiation, particularly using Nd:YAG (neodymium-doped yttrium aluminum garnet) lasers. These lasers effectively roughen zirconia surfaces, improving micromechanical retention. 11 13 Nd:YAG lasers are widely used in dentistry due to their specific wavelength and absorption characteristics. 14 Additionally, argon laser cleaning plasma has been shown to enhance shear bond strength (SBS), although results depend on the duration of application. 14

Another alternative is cold atmospheric plasma (CAP), which improves surface wettability without altering the material's structure. 7 9 15 16 CAP enhances bond strength by reducing carbon contamination 8 and increasing surface energy, 8 9 allowing for better adhesive penetration. Unlike APA, CAP does not induce mechanical stress, meaning it preserves the zirconia's crystalline phase and microstructure. 7 8 9 Beyond surface pretreatments, selecting the right bonding protocol is crucial. The incorporation of phosphate-ester monomers like 10-methacryloyloxydecyl dihydrogen phosphate (10-MDP) improves chemical adhesion between zirconia and resin by enhancing wettability. 17 18

The conventional and updated surface pretreatment methods, bonding protocols, and different monolithic zirconia materials have been evaluated separately or in combination in previous studies. Consequently, this study aimed to compare all these factors within a single experiment, thereby facilitating a more comprehensive evaluation and yielding less heterogeneous conclusions. The primary objective of the present study was to assess the impact of laser versus various surface pretreatment protocols on the SBS of two distinct monolithic zirconia materials using two different luting cement protocols. The null hypotheses were as follows: (1) There is no significant difference in SBS among the different zirconia surface pretreatments. (2) The two monolithic zirconia materials (3Y-TZP and 5YSZ) do not exhibit significantly different SBS. (3) The bonding protocols do not influence the SBS values.

## Materials and Methods

### Study Design and Sample Size Calculation

The sample size for this study was calculated to evaluate the effect of five different surface pretreatments (control vs. four experimental groups) each used with/without primer, on the SBS of two types of zirconia (3Y-TZP and 5YSZ) with composite. Using the G*Power statistical power analysis program (version 3.1.9.4) for sample size calculation, a total of 180 specimens (9 in each subgroup) were determined to be adequate for detecting a large effect size ( *f* ) of 0.51, achieving an actual power (1– *β* error) of 0.8 (80%) and a significance level ( *α* error) of 0.05 (5%) for a two-sided hypothesis test. 19 To enhance the study's statistical power, 20 final subgroups were assigned ( *n* = 10) based on surface pretreatment, adhesion protocols, and yttria contents, with 200 specimens ( *n* = 100 based on yttria contents).

### Preparation of Zirconia Specimens

A total of 200 square zirconia specimens were prepared from zirconia blanks with different yttria content: 100 specimens of 3Y-TZP (ceraMotion Z HT Multishade) (Dentaurum, Germany), and 100 5YSZ (ceraMotion Z Cubic Multishade) (Dentaurum, Germany). The blanks were wet cut into discs using a precision cutting machine (IsoMet 4000 microsaw, Buehler, Illinois, United States). 20 A 0.6-mm diamond disc was used with 2,500 revolutions per minute speed and a 10-mm/min feed rate. The specimens were then wet ground using a 600-grit SiC on an Automet 500 (Buehler, Esslingen, Germany), followed by ultrasonic cleaning and drying. 21 The specimens were sintered in a Sirona inFire HTC speed furnace (Sintering Furnace, Sirona, Germany) following the manufacturer's instructions, resulting in 10 × 10 × 2.5 mm square-shaped specimens. Finally, tested specimens were ultrasonically cleaned in 99% isopropanol for 3 minutes.

### Composite Sample Fabrication

A specially designed mold was developed to standardize the dimensions and positions of 200 composite specimens. This mold consists of a square Teflon base featuring a central cavity measuring 10 × 10 × 2.5 mm, intended to accommodate zirconia specimens. Additionally, a circular split Teflon mold with an inner split square (6 mm ^2^ surface area, 4 mm in height) is positioned directly above the square mold to contain the composite material. An outer stabilizing ring was employed to secure the molds together.A transparent glass slab was placed beneath the molds, and light-polymerized nanohybrid composite resin (Tetric N-ceram, Ivoclar Vivadent AG, Liechtenstein) was applied in circular mold in multiple 2 mm increments. The resin was densely packed with a plastic instrument and polymerized with a halogen light (Elipar, 3M ESPE, Leicestershire, England) at 1,350 mW/cm ^2^ for 40 seconds per increment. After completion, composite specimens were detached and polymerized for 20 seconds, and excess composite was removed with a micromotor. Visual inspection ensured defect-free specimens, which were then calibrated for standardization.

### Surface Pretreatment of Zirconia Specimens

**Fig. 1:**
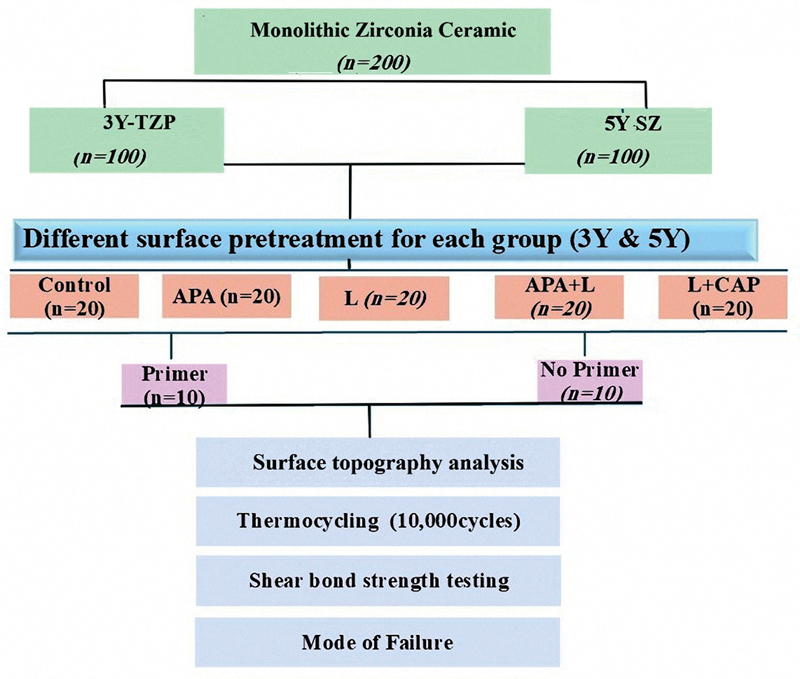
Diagram representing all steps of the study.

Each zirconia main group ( *n* = 100 for 3Y-TZP, and *n* = 100 for 5YSZ) was randomly subdivided into five subgroups ( *n* = 20) based on surface pretreatments as follows: No surface pretreatments as a control (C), APA, Nd:YAG laser irradiation (L), combination of APA and laser irradiation (APA + L), and finally combination of laser irradiation and CAP (L + CAP), Fig. 1 .

**Fig. 2:**
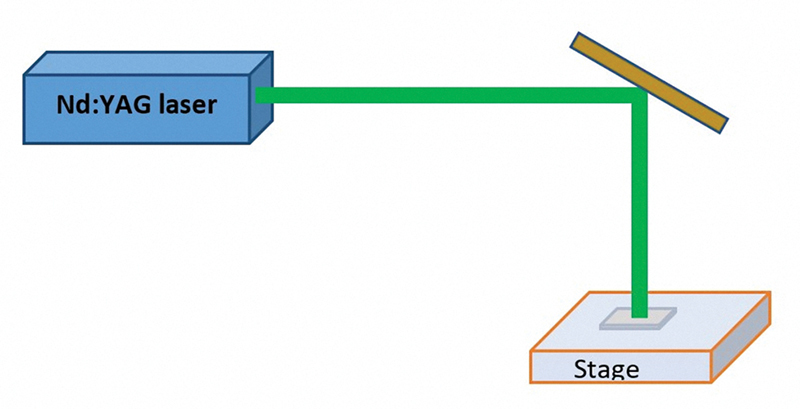
Diagram showing neodymium-doped yttrium aluminum garnet (Nd:YAG) laser irradiation surface treatment.

(1) For control group (C): No surface pretreatment.
(2) For APA: Zirconia surfaces were abraded using 50 µm aluminum oxide (Al2O3) particles (Korox 50, BEGO, Bremen, Germany) for 20 seconds at a pressure of 2 bar, with the particles applied perpendicularly from a distance of 10 mm. 16 To ensure methodological consistency, a custom three-part holder was employed, consisting of a wooden base to support the zirconia specimen, a metal ring to securely fasten the microblaster with screws, and a metal arm designed to maintain a perpendicular alignment between the nozzle and the specimen.
(3) Nd:YAG laser irradiation (L): The bonding surfaces were treated with an Nd:YAG laser (Smart 2940D Plus, Deka Laser, Florence, Italy) operating at 532 nm in its second harmonic generation. The laser had an 8-mm beam diameter, 10 nanoseconds pulse width, and 10 Hz frequency, with an output power of 200 mJ and a 3-minute exposure time, Fig. 2 . 22
(4) Combination of APA and Nd:YAG laser irradiation (APA + L): Zirconia surfaces were air-abraded with 50 µm aluminum oxide (Al2O3) particles at 2 bar pressure from a 10-mm distance for 20 seconds, followed by 3 minutes of Nd:YAG laser irradiation.
(5) Combination of Nd:YAG laser irradiation and CAP (L + CAP): Specimen bonding surfaces were irradiated with an Nd:YAG laser for 3 minutes, followed by 5 minutes of CAP pretreatment using a dielectric barrier discharge air plasma system operating at atmospheric pressure. 23 The upper electrode, powered by an AC high-voltage system (Plasma Driver PVM500, Information Unlimited Co.), delivered up to 20 kV and a 20-kHz sinusoidal signal, with a 33.33 kΩ current-limiting resistor in place. Treated specimens were positioned on a grounded lower electrode during CAP exposure.

### Scanning Electron Microscopy

Two extra specimens were added to each group for this analysis and then randomly selected before the assessment. After surface pretreatment, the topography of specimens from each group was analyzed using an environmental scanning electron microscope (Thermo Fisher Scientific Inc., United States) at 2,500× magnification and 30 kV accelerating voltage. Image contrast was enhanced with Thermo Scientific Maps software.

### Adhesive Cementation Procedure

For half of each subgroup (control and four experimental groups, *n* = 100), zirconia primer (Z-Prime Plus; BISCO, Schaumburg, Illinois, United States) was applied for 10 seconds, air-dried, and followed by cementation of zirconia and composite disks using self-adhesive resin cement (Maxcem Elite Chroma, Kerr Corporation, Italy). The specimens were secured under a 0.2-kg constant load to ensure uniform cementation, 24 with excess cement removed using foam pellets, and margins light-polymerized for 40 seconds per side.

The other half ( *n* = 100) underwent the same cementation process without primer application. All specimens were stored in distilled water at 37°C for 24 hours before thermocycling.

### Thermocyclic Aging of Adhesive Assembly Specimens

The adhesive assembly specimens were stored in distilled water for 24 hours and underwent thermocycling for 10,000 cycles (SD Mechatronic, Thermocycler, Westerham, Germany). The temperature changed between 5°C and 55°C baths, with a dwell time of 30 seconds and a transfer time of 10 seconds. 21

### Shear Bond Strength Testing

A universal testing machine (Instron, England) with a mono-beveled chisel rod was used to evaluate zirconia/composite specimens. A 5-kg load was applied at a crosshead speed of 0.5 mm/min, and the debonding force was recorded in Newtons. SBS ( *τ* ) in MPa was calculated using the formula: *τ* = *F* / *A* , where *τ* is the SBS in MPa, *F* is a force at failure, and *A* is the bonding area in mm ^2^ . Data was recorded and processed using the BlueHill software (Universal Instron, England).

### Mode of Failure Analysis

A stereomicroscope (Nikon SMZ745T, Japan) operating at 30× magnification was employed to evaluate the failure modes based on the following assortments: (1) adhesive, indicating separation of the resin or cement from the zirconia, (2) cohesive in the cement or resin, signifying fracture occurring solely within these materials, and (3) mixed, characterized by residual bits of resin cement partially visible on the zirconia surface.

### Statistical Analysis

Statistical analysis was conducted using SPSS software (SPSS 20, SPSS, Inc., Chicago, Illinois, United States). Numerical data were summarized as mean, standard deviation, confidence intervals, and range. Data normality was assessed using distribution checks, Kolmogorov–Smirnov, and Shapiro–Wilk tests. One-way analysis of variance (ANOVA) with Bonferroni's post hoc test was used for group comparisons, while the independent *t* -test evaluated intragroup differences. A multiway ANOVA analyzed interactions between variables. A significance level of *p* ≤ 0.05 was set.

## Results

1. Scanning electron microscopy (SEM) evaluation

**Fig. 3:**
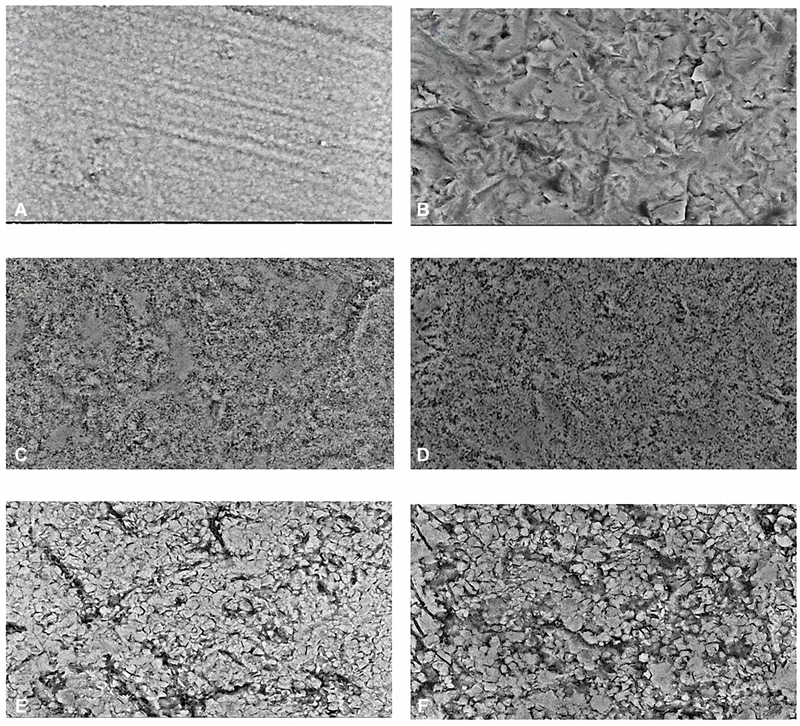
Scanning electron microscope images (×2,500) showing the effect of different surface pretreatments on randomly selected specimens, ( **A** ) control, ( **B** ) air-particle abrasion (APA), ( **C** ) laser irradiation (L), ( **D** ) L + cold atmospheric plasma (CAP), and ( **E** and **F** ) APA + L.

SEM analysis of the randomly selected specimens from the zirconia groups revealed different surface topographical features. The control group exhibited the primary microstructural appearance of zirconia, characterized by parallel demarcations, which may be attributed to the refining process conducted with an Automet device and sandpapers, resulting in these parallel markings. The APA specimens displayed highly irregular and rough surfaces, with micropores that likely correspond to the sharp edges of the alumina particles. In contrast, the laser surface pretreatment resulted in more uniform nanoscale changes, presenting a more mosaic appearance than the APA group. The degree of surface roughening was not significantly pronounced, as the finishing demarcations remained visible on the surface. The group subjected to both laser pretreatment and CAP exhibited a surface texture similar to that of the laser group. Conversely, the combination of APA and laser pretreatment yielded the most pronounced roughness among all groups, characterized by irregular surfaces akin to the APA group; however, this combination featured deeper microfissures surface cracks and more protruded, less embedded zirconia particles, as depicted in Fig. 3 .

2. Shear bond strength

**Fig. 4:**
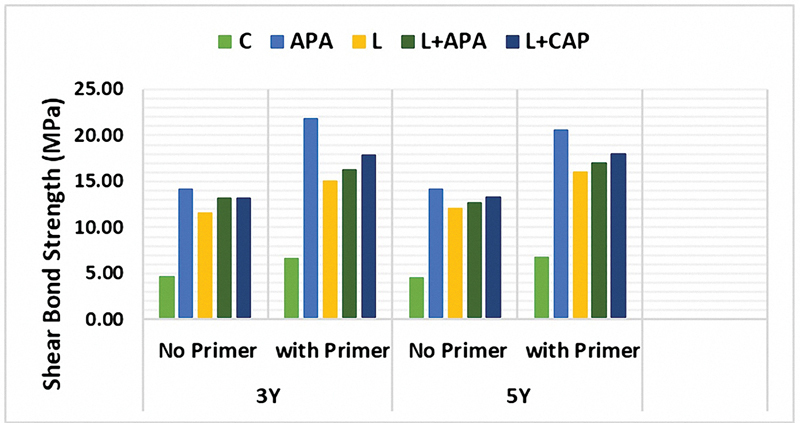
A column chart illustrating the mean of shear bond strength (MPa) to composite after different surface pretreatments.

Different surface pretreatments exhibited significant differences among the groups ( *p* < 0.001) as follows: APA demonstrated a statistically significantly higher SBS than all other groups, followed by APA combined with laser pretreatment. Laser pretreatment alone, as well as its combination with CAP, ranked second after the APA groups ( *p* < 0.001), with a slight enhancement of SBS noted following CAP application to a laser-treated surface. Conversely, the control group exhibited very low SBS. The two types of monolithic zirconia did not demonstrate a statistically significant difference in SBS ( *p* > 0.05) when comparing each 3Y-TZP and 5YSZ group subjected to the same surface pretreatment and adhesion protocol. Furthermore, a statistically significant increase in SBS was observed with the application of a zirconia primer ( *p* < 0.001). Comparisons among the different groups and their interactions are presented in Table 1 . Detailed descriptive statistics and comparisons among the different groups and their interactions are presented in Table 2 and Fig. 4 .

**Table 1:** Mean and standard deviation with statistical differences of different surface treatments and bonding protocols

Material	Surface treatment	Bonding protocol				Significance
		No primer		Primer		
		Mean	Standard deviation	Standard deviation	Standard deviation	
3Y-TZP	C	4.71 ^Bc^	0.96	6.78 ^Ad^	1.03	*p* < 0.001 and *p* > 0.05
	APA	14.20 ^Ba^	2.21	21.90 ^Aa^	3.07	
	L	11.71 ^Bb^	1.60	15.11 ^Ac^	1.42	
	L + CAP	13.24 ^Ba,b^	0.77	16.37 ^Ab,c^	1.08	
	APA + L	13.29 ^Ba,b^	0.93	17.99 ^Ab^	1.35	
Significance		*p* < 0.001 and *p* > 0.05		*p* < 0.001 and *p* > 0.05		
5YSZ	C	4.62 ^Bc^	1.04	6.86 ^Ad^	0.84	*p* < 0.001 and *p* > 0.05
	APA	14.26 ^Ba^	1.59	20.72 ^Ab^	1.88	
	L	12.15 ^Bb^	1.00	16.06 ^Ac^	1.64	
	L + CAP	12.82 ^Bb^	0.67	17.15 ^Ab,c^	1.06	
	APA + L	13.36 ^Ba,b^	1.03	18.03 ^Aa^	2.18	

Abbreviations: APA, air-particle abrasion; C, control; CAP, cold atmospheric plasma; L, laser irradiation.

Note: Uppercase letters indicate the statistically significant levels within the row. Lowercase letters indicate statistically significant values within the column.

**Table 2:** Results of multiple-way ANOVA test for the interaction of variables

Source	Type III sum of squares	df	Mean square	*F*	*p* -Value	Partial eta squared	Observed power
Surface pretreatment (ST)	3326.39	4.00	831.60	375.98	0.0001 a	0.893	1.00
Primer application (P)	907.09	1.00	907.09	410.11	0.0001 a	0.695	1.00
Type of Zr material (3Y and 5Y) (ZrM)	0.27	1.00	0.27	0.12	0.727ns	0.001	0.06
ST × P	104.61	4.00	26.15	11.82	0.0001 a	0.208	1.00
ST × ZrM	60.68	4.00	15.17	6.86	0.0001 a	0.132	0.99
P × ZrM	0.19	1.00	0.19	0.08	0.772ns	0.000	0.06
ST × P × Zr	60.48	4.00	15.12	6.84	0.0001 a	0.132	0.99

Abbreviations: df, degree of freedom; ns, nonsignificant; P, primer application; ST, surface pretreatment; Zr, zirconia; ZrM, Zr material.

Note: Significance level *p* ≤ 0.05.

a Significant.

3. Mode of failure

**Fig. 5:**
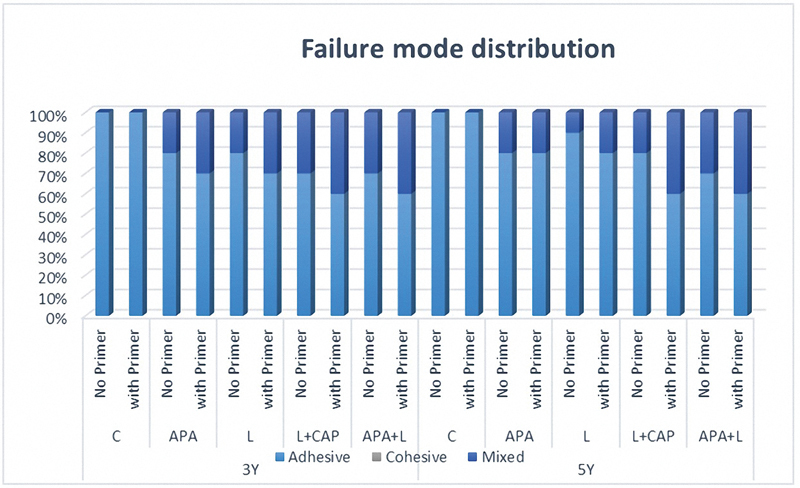
A stacked column chart showing failure mode distribution of 3Y and 5Y zirconia.

The failure mode observed in all tested groups was exclusively adhesive and mixed failures. None of the luted specimens exhibited cohesive failures. In the control groups, all failure modes were classified as adhesive failures. In the groups subjected to surface pretreatment, both adhesive and mixed failures were documented, as illustrated in Fig. 5 .

## Discussion

This study evaluated the effects of various surface pretreatment and adhesive luting protocols on the SBS of two monolithic zirconia materials, specifically 3Y-TZP and 5YSZ. The first null hypothesis was rejected, indicating that different surface pretreatments significantly influenced SBS. The second null hypothesis, which posited that the two types of monolithic zirconia would not exhibit different SBS values, was partially rejected; no significant effect of varying yttria content on SBS was observed in all groups, except the APA group with zirconia primer, 3Y-TZP showed higher SBS values than 5YSZ when utilizing the same APA pretreatment and primer surface treatment. While the 5YSZ group pretreated with APA and L exhibited statistically higher SBS than 3Y-TZP with the same surface pretreatment and surface treatment. The third null hypothesis, which suggested that different bonding protocols would have no impact on SBS values, was rejected, 10-MDP primer surface treatment enhanced SBS values in all groups, irrespective of the surface pretreatment applied.

It is well established that zirconia restorations require pretreatment of their intaglio or adhesive surfaces to ensure long-term survival. This study strongly corroborated this assertion, as untreated groups demonstrated markedly lower SBS compared with the treated groups. 25 Additionally, applying APA resulted in the highest SBS among all groups. APA has been utilized for decades to prepare zirconia surfaces, with bond strength consistently reported as reliable and durable. 2 5 Although previous studies have indicated that APA may have detrimental effects on zirconia surfaces, such as inducing premature phase transformations and microcracks, its efficacy remains as reliable as it has been observed over the years.

The Nd:YAG laser represents a novel mechanical pretreatment technique that facilitates precise control over surface modifications. This method effectively enhances the surface roughness of zirconia substrates following pretreatment. The Nd:YAG laser can achieve zirconia surface roughness comparable to that produced by APA, without adversely affecting the zirconia microstructure. 26 A decade ago, a study indicated that surface pretreatments of zirconia using Nd:YAG laser did not yield any significant benefits; even with increased irradiation power and prolonged exposure time, the bond strength of zirconia remained unaltered. 27 In the current study, SBS was significantly superior in the APA group; however, it remained within a similar range when compared with Nd:YAG-treated specimens. This finding is corroborated by SEM images, which demonstrated that APA surface pretreatments resulted in greater depth of roughness due to the abrasion caused by aluminum oxide particles, potentially elucidating the higher SBS values observed in the APA group. The superior performance of APA specimens in terms of SBS is consistent with previous research comparing APA to Nd:YAG surface pretreatments, with observed differences mirroring those found in the present study. 28 Although the results of the current study indicated elevated SBS for both the APA and Nd:YAG groups, these outcomes may be attributable to procedural variations. Nevertheless, the findings of all groups with surface pretreatment reveal a significant enhancement in zirconia SBS when treated with zirconia MDP containing primer and bonded to self-adhesive resin cement and composite specimens. These results are consistent with prior studies, 29 30 31 reinforcing the efficacy of laser pretreatment in improving bonding performance. A study conducted by Akin et al 30 demonstrated that optimizing erbium-doped:YAG (Er:YAG) laser parameters—specifically, an energy setting of 150 MJ, a frequency of 10 Hz, and a pulse duration of 20 seconds—significantly enhances the SBS of Y-TZP surfaces. Additionally, another study reported comparable SBS values for APA, photodynamic therapy, and irradiation using both Nd:YAG and Er:YAG lasers. 32 Furthermore, bonding of 3Y-TZP and 5YSZ to dentin represented higher SBS for APA followed by laser and CAP, with significant increase in bond strength by far with the application of 10-MDP containing primer. 33

The combination of Nd:YAG laser pretreatment with APA surface pretreatment demonstrated a reduction in SBS across all primary groups when APA pretreatment was applied independently. Although the outcomes for the APA and APA + Nd:YAG groups were within the same range. Nevertheless, these combinations may adversely affect SBS. This phenomenon may be attributed to the laser's capacity to increase further the roughness of surfaces already modified by APA, resulting in the attenuation of the irregularities created by APA. Furthermore, the laser pretreatment may excessively increase surface roughness so that zirconia grains are not sufficiently embedded within the matrix. Thereby facilitating the slightly earlier detachment of the cement-zirconia assembly, resulting in a slight reduction of SBS compared with APA surface pretreatment alone.

CAP is a partially ionized gas that enhances surface energy and hydrophilicity by generating reactive species, including ions, electrons, and free radicals. Research has demonstrated its effectiveness in modifying the surfaces of dental ceramics and natural teeth, thereby increasing hydrophilicity. 34 The current study indicates that the combination of inert surface pretreatments, specifically the Nd:YAG laser followed by CAP, resulted in a greater SBS enhancement than the application of the Nd:YAG laser alone. Notably, both pretreatment protocols significantly increased SBS compared with the untreated control specimens. These findings are consistent with the study conducted by Kamiş and Eser, 35 which reported that surface pretreatment with APA or the combination of laser and CAP enhances SBS, suggesting that CAP improves the wettability and bond affinity of zirconia surfaces, particularly in conjunction with the application of 10-MDP phosphate monomer. 35

The benefits of zirconia primer application in this study are attributed to incorporating a 10-MDP component within the applied material, as demonstrated by numerous preceding laboratory studies 2 6 and clinical investigations. 1 2 5 Applying zirconia primer facilitates chemical interactions that enhance bond strength. This phenomenon was clearly illustrated in the current research, where SBS values significantly increased, independent of the surface pretreatment or the material utilized. Comparable findings have been documented in earlier laboratory studies and meta-analyses. 25 36

The mode of failure observed in this study demonstrated complete adhesive debonding at the surface of zirconia when left untreated, thereby highlighting the critical significance of surface pretreatment in achieving adequate microroughness. The groups subjected to surface pretreatment exhibited an increase in mixed failure modes that correlated with the enhanced SBS, characterized by remnants of self-adhesive resin cement adhered to the treated zirconia surfaces. This particular type of mixed failure aligns with findings from prior studies, which associate elevated bond strength values with increased mixed and cohesive failure modes. 6 37

To simulate the oral environment and assess bond strength, thermal cycling comprising 10,000 cycles, approximately equivalent to 1 year, was performed. 21 However, it is important to note that no mechanical aging, artificial saliva, or beverages were utilized during the aging process. Furthermore, the study employed only one type of luting cement and two ceramic materials from the oxide ceramic family. Despite these limitations, the advantages of the bonding protocol utilizing the 10-MDP primer and surface pretreatments with APA, laser, cold plasma, or the combination of APA and laser were identified. These findings warrant further investigation under prolonged thermomechanical aging conditions with significant loading on natural teeth or clinical settings.

## Conclusion

Within the limitations of this study, it can be concluded that:

1. The highest SBS values were observed in the APA groups.
2. Laser surface pretreatment has been shown to enhance the bond strength of zirconia to composite materials when luted with self-adhesive resin cement.
3. The combination of laser with APA or CAP pretreatments results in a slight reduction in SBS.
4. The application of phosphate monomer, specifically 10-MDP, significantly enhances SBS.
5. Different zirconia materials with 3 and 5 mol% yttria contents demonstrated no significant differences in SBS; thus, no special considerations need to be taken.
